# Psychiatric manifestations of lipoid proteinosis with temporal lobe involvement: A case report

**DOI:** 10.1192/j.eurpsy.2026.11068

**Published:** 2026-07-31

**Authors:** H. E. Baki

**Affiliations:** Department of Psychiatry, Fatsa State Hospital, Ordu, Türkiye

## Abstract

**Introduction:**

Lipoid proteinosis (Urbach-Wiethe disease) is a rare autosomal recessive disorder caused by *ECM1* mutations. In patients with medial temporal lobe calcifications, neuropsychiatric conditions, including anxiety, mood, and psychotic disorders, as well as impairments in memory, emotion recognition, and executive functions, have been described. However, longitudinal descriptions of psychiatric manifestations and treatment responses are rarely reported.

**Objectives:**

To describe the longitudinal psychiatric manifestations and treatment response in a patient with lipoid proteinosis and medial temporal lobe calcification, and to contextualize these observations with a brief narrative review.

**Methods:**

The report was prepared in accordance with international CARE guidelines. Data were obtained from clinical records, including psychiatric and neurological assessments, EEG, brain imaging, and histopathological findings.

**Results:**

The patient is a 24-year-old woman with lifelong hoarseness and mucocutaneous lesions. At 18, a lower-lip mucosal biopsy showed PAS-positive, eosinophilic hyaline material in the papillary dermis, confirming the diagnosis of lipoid proteinosis. In the same year, she presented with performance anxiety, stress-related presyncopal episodes, concentration difficulties, and forgetfulness. She was diagnosed with an anxiety disorder and treated with fluoxetine 20 mg/day, with symptomatic improvement. Two years prior to admission, she experienced a brief convulsive event (15–20 s), with loss of consciousness and tonic spasms. EEG showed intermittent focal temporal slowing without definite epileptiform discharges, and brain CT revealed a calcification in the right medial temporal lobe (Image 1). In the following months, she developed recurrent paroxysmal panic attacks with palpitations, shortness of breath, and intense fear. She also had emotional dysregulation with sudden crying spells and affective lability. Sertraline was initiated and titrated to 100 mg/day. Panic attack frequency decreased, though attentional and organizational difficulties persisted, so methylphenidate 20 mg/day was added. Over 10 months of follow-up, daily functioning improved with reduced emotional dysregulation and no recurrence of seizure-like episodes or panic attacks.

**Image 1:**
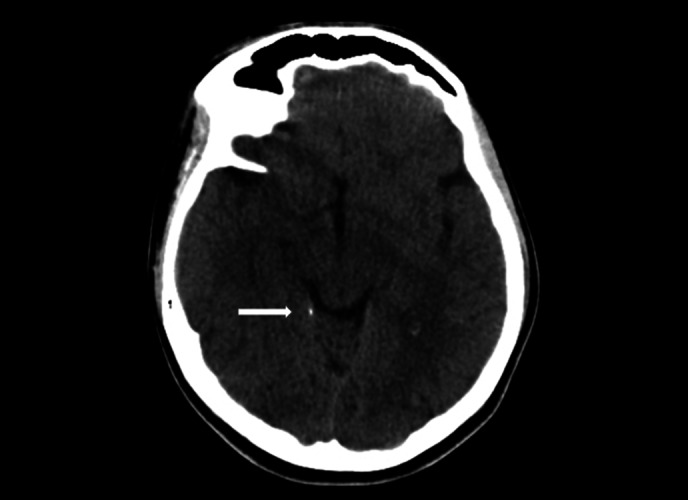

**Conclusions:**

This case highlights the heterogeneity of neuropsychiatric symptoms in lipoid proteinosis. Unlike the predominantly bilateral calcifications reported in the literature, our patient presented with a unilateral medial temporal lesion. Medial temporal lobe involvement may provide the neurobiological substrate for paroxysmal panic attacks. Sertraline alleviated panic and anxiety symptoms, while methylphenidate improved executive dysfunction, underscoring the potential role of standard psychiatric treatments in this rare disorder. Further documentation of the psychiatric course in lipoid proteinosis will help refine clinical management.

**Disclosure of Interest:**

None Declared

